# Synergistic Regulation of Interfacial Potential and Anionic Covalency for High‐Voltage Cobalt‐Free All‐Solid‐State Batteries

**DOI:** 10.1002/anie.9550345

**Published:** 2026-06-04

**Authors:** Yue Wang, Shuibin Tu, Long Qian, Shijie Xu, Chao Ye, Shi‐Zhang Qiao

**Affiliations:** ^1^ School of Chemical Engineering Adelaide University Adelaide SA Australia

**Keywords:** cobalt‐free, high‐voltage all‐solid‐state batteries, interfacial oxygen chemistry, LiNi_0.5_Mn_1.5_O_4_ cathode, space charge layer

## Abstract

High‐voltage cobalt‐free all‐solid‐state lithium batteries (ASSLBs) represent a promising pathway toward high‐energy‐density and sustainable energy storage. However, their practical viability is fundamentally hindered by a coupled interfacial failure mechanism involving kinetic bottlenecks at the space‐charge layer (SCL) and the electrochemical instability of interfacial lattice oxygen. Here, we propose a synergistic regulation to decouple these constraints in 5 V‐class LiNi_0.5_Mn_1.5_O_4_ (LNMO) ASSLBs. We reveal that the large lithium (Li) chemical potential mismatch at the LNMO/electrolyte interface drives a Li‐deficient SCL, while the high voltage triggers interfacial oxygen release, causing severe interfacial structural degradation. To address this, a stable interface was constructed where interfacial potential and anion covalency are regulated synergistically. Specifically, a high‐dielectric BaTiO_3_ (BTO) coating layer was introduced to regulate interfacial potential and suppress SCL formation, while sulfate‐derived S─O covalent bonds stabilized the interfacial lattice oxygen. Consequently, the BTO‐S‐LNMO ASSLB achieves a notable increase in reversible capacity from 52 to 116 mAh g^−1^ at 0.1 C and enables high‐rate capacity up to 3 C and exhibits long‐term durability at 1 C. This work establishes a paradigm of coupling dielectric regulation and anion‐chemistry stabilization to unlock the potential of high‐voltage LNMO ASSLBs.

## Graphical abstract

A synergistic interface regulation strategy using high‐dielectric BaTiO_3_ and sulfate‐derived S─O bonds is developed to unlock high‐voltage LiNi_0.5_Mn_1.5_O_4_ all‐solid‐state batteries. By mitigating space‐charge layer effects and stabilizing lattice oxygen, the system achieves high‐rate (3 C) and durable operation in 5 V‐class cobalt‐free solid‐state batteries.

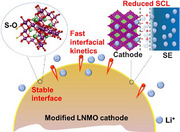

## 1 Introduction

All‐solid‐state lithium batteries (ASSLBs) have emerged as the next‐generation energy storage technology due to their high energy density and intrinsic safety [1, 2]. In recent years, layered ternary oxide cathodes (LiMO_2_, M = Ni, Co, Mn, Al, etc.) have demonstrated competitive performances in ASSLBs, offering high capacity and extended lifespans [3, 4]. However, the high cost and supply chain uncertainty of cobalt raise the concerns regarding cost volatility and long‐term sustainability [5, 6]. In contrast, cobalt‐free spinel‐phase LiNi_0.5_Mn_1.5_O_4_ (LNMO) cathodes offer advantages such as cost‐effectiveness, a high out‐put potential of ∼4.7 V vs. Li/Li^+^, and a theoretical energy density of >650 Wh kg^−1^ with a specific capacity of 147 mAh g^−1^, making them appealing candidates for high‐energy‐density ASSLBs [7, 8, 9].

Among current solid electrolyte (SE) candidates, sulfide and halide SEs are regarded as two of the most promising systems for ASSLBs due to their high ionic conductivities. However, Jang et al. have demonstrated that, for high‐voltage LNMO cathode, sulfide SEs such as Li_6_PS_5_Cl (LPSCl) suffer from intrinsic chemical incompatibility, leading to severe SE decomposition and rapid interfacial degradation, thereby highlighting the necessity of using halide SEs with higher oxidative stability limits (∼4 V vs. Li/Li^+^) [10]. Benefiting from wide oxidative stability and good interfacial compatibility with LiCoO_2_ and LiMO_2_ cathodes, halide SEs have recently been regarded as promising SEs for high‐voltage cathodes [11, 12, 13]. Nevertheless, when directly paired with the high‐voltage LNMO cathode, continuous interfacial side reactions still occur under the extreme operating voltage of LNMO [14]. To address this, considerable efforts have been devoted to developing interfacial coatings on LNMO cathodes such as Li_3_PO_4_ and LiNbO_3_ [15, 16, 17, 18, 19, 20], which improve interfacial compatibility between LNMO and halide SEs. However, the electrochemical performances of current LNMO ASSLBs are still unsatisfactory, typically limited to fewer than 100 cycles at low current densities < 0.1 C. Consequently, long‐term cycling of LNMO in ASSLBs under high‐rate conditions has yet to be realized.

These performance bottlenecks primarily originate from the formation of the space‐charge layer (SCL) under the high operating voltage at the LNMO/SE interface. The formation of SCL results from spontaneous Li‐ion migration from the SE to the cathode driven by a pronounced lithium chemical potential mismatch [21, 22]. The depletion of the Li‐ion concentration at the SE side leads to a high interfacial resistance that significantly impedes ion transport. More importantly, the high operating potential of LNMO triggers oxygen‐related parasitic reactions with the SE, generating electrochemically inactive interphases that further hinder interfacial ion transport. However, current understanding of interfacial failure mechanisms governed by coupled degradation factors in LNMO ASSLBs remains qualitative, and the intrinsic origins of these side reactions are not fully elucidated. Deciphering the mechanism of SCL formation and interfacial parasitic reactions is therefore critical to engineering the stable microstructure required for high voltage LNMO ASSLBs.

In this work, we proposed a synergistic regulation strategy to simultaneously address transport constraints and interfacial degradation via potential modulation and anion‐chemistry stabilization. Specifically, a piezoelectric BaTiO_3_ (BTO) layer was introduced on the LNMO surface to establish a built‐in electric field that suppresses SCL formation at the LNMO/Li_3_InCl_6_ (LIC) interface. More importantly, we identify that the instability of surface lattice oxygen in LNMO at high potentials is highly related to the interfacial parasitic reactions with LIC. To stabilize these lattice oxygen, strong covalent S─O bonds were constructed via a sulfidation treatment. These S─O bonds weaken the local transition metal‐oxygen (TM–O) hybridization, thereby suppressing oxygen‐induced interfacial degradation and maintaining a stable interfacial microstructure during cycling. Consequently, ASSLBs with BaTiO_3_‐S‐LNMO (BTO‐S‐LNMO) cathodes deliver a high specific capacity of 116 mAh g^−1^ at 0.1 C, significantly surpassing that of pristine LNMO (52 mAh g^−1^). Moreover, the high‐rate capability of LNMO‐based ASSLBs is demonstrated for the first time, while previous studies were largely limited to ∼0.1 C, the BTO‐S‐LNMO cathode delivers 66.8 mAh g^−1^ at 3 C and maintains stable performance over 200 cycles at 1 C. This study provides fundamental mechanistic insights into the interfacial degradation of high‐voltage LNMO cathodes and proposes a synergetic strategy combining interfacial potential modulation with oxygen stabilization to enable high‐energy LNMO‐based solid‐state energy storage.

## 2 Results and Discussion

### 2.1 Interfacial Degradation Mechanisms of LNMO ASSLBs

**FIGURE 1:**
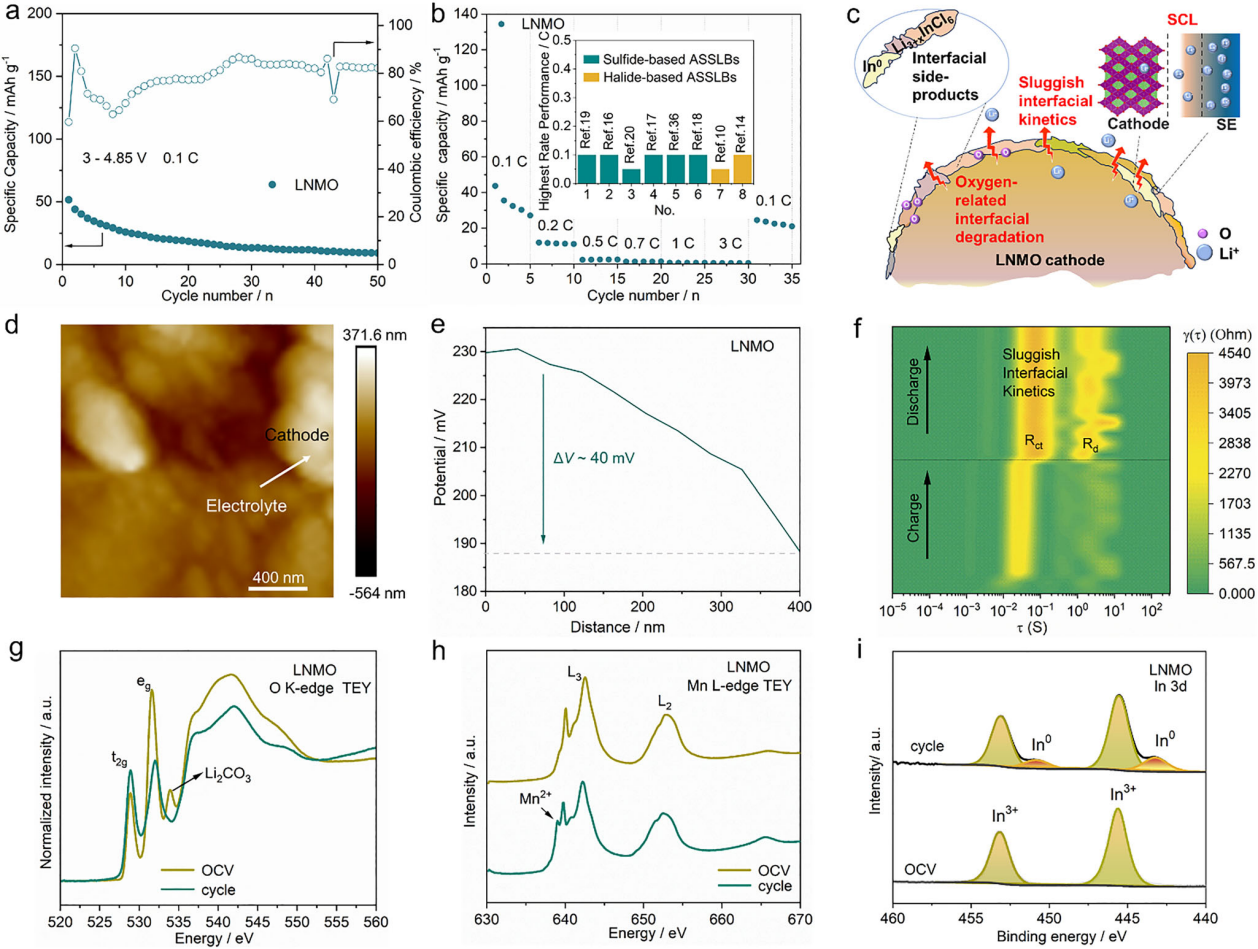
Inferior electrochemical performance and associated degradation mechanisms in LNMO ASSLBs. (a) Cycling performances at a rate of 0.1 C and (b) rate capability of LNMO ASSLBs. The inset shows current densities from previously reported literature. (c) Schematic illustration of the degradation mechanism of LNMO ASSLBs. (d) AFM images of the LNMO composite cathode. (e) Surface potential distribution from LNMO to LIC by KPFM along the direction indicated by the straight line with arrows in (d). (f) DRT profile transformation derived from GEIS during the first cycle of LNMO ASSLBs. (g) O K‐edge and (h) Mn L‐edge for the LNMO composite cathodes before and after 50 cycles in the TEY mode. (i) In 3d XPS spectra of the LNMO/LIC composite cathodes before and after 50 cycles.

To investigate the origins of the limited capacity and rate capability in high‐voltage LNMO cathodes, the ASSLBs were assembled utilizing pristine LNMO as the cathode, halide LIC as the SE (with ionic conductivity of 1.18 mS cm^−1^, Figure S1), and a lithium–indium (Li–In) alloy as an anode. As shown in Figure 1a, the ASSLBs with pristine LNMO cathodes exhibit a low initial capacity of 52 mAh g^−1^ at 0.1 C and suffer from rapid capacity fading thereafter, verifying the inferior electrochemical reversibility. Meanwhile, the Coulombic efficiency (CE) of LNMO ASSLB remains at around 80% throughout the cycling, implying severe interfacial parasitic reactions. Furthermore, the LNMO‐based ASSLBs demonstrate compromised rate capability, delivering 2.2 mAh g^−1^ at 0.5 C and negligible capacity at higher current densities (Figure 1b). Notably, previously reported charge/discharge rates for LNMO‐based ASSLBs are generally restricted to ≤ 0.1 C (inset in Figure 1b), highlighting kinetic limitations of this system. Such inferior electrochemical performance indicates pronounced interfacial incompatibility and degradation. Generally, the interfacial degradation of high‐voltage cathodes in ASSLBs can be attributed to the formation of the SCL layer at the cathode/SE interface and the severe interface parasitic reaction, as depicted in Figure 1c. These issues are significantly exacerbated by the high operating potential (> 4.7 V vs. Li/Li^+^) of LNMO, ultimately resulting in poor cycling and rate performance.

To elucidate the SCL formation at the LNMO/LIC interface, atomic force microscopy combined with Kelvin probe force microscopy (AFM‐KPFM) was employed to map the surface potential distribution across the LNMO and LIC in the composite cathodes [23]. As shown in Figure 1d, the interfacial potential profile crossing from the LNMO cathode to the LIC SE was recorded as a white line in Figure 1e. The resulting interfacial potential drop from ∼230 mV at the cathode surface to ∼188 mV at the SE surface, corresponding to a high interfacial potential difference (ΔV) of ∼42 mV at the LNMO/LIC interface. In addition, histograms of interfacial potential distribution obtained from bearing analysis reveal a broad potential variation of ∼62 mV at the LNMO/LIC interface (Figure S2), confirming an inhomogeneous interfacial charge distribution and the formation of SCL. This significant potential barrier at the LNMO/LIC interface severely hinders Li^+^ migration, thereby compromising the battery reproducibility. The initial charge–discharge curve further confirms the formation of SCL. As shown in Figure S3, a notably sluggish slope below 4 V is observed during initial charging, which arises from the formation of SCL at the LNMO/LIC interface [24]. Moreover, the significantly shortened charging plateau at ∼4.7 V (vs. Li/Li^+^) suggests suppressed Ni redox reaction [25, 26], resulting in a low initial specific capacity and a low initial CE of only 59.63%. The cyclic voltammetry (CV) results further confirm the suppressed Ni redox (Figure S4), demonstrating that the SCL largely impedes the interfacial Li^+^ migration, therefore lowering the reversibility of LNMO ASSLBs.

To probe the dynamics of Li^+^ transportation at the LNMO/LIC interface, in situ galvanostatic electrochemical impedance spectroscopy (GEIS) was conducted to track the impedance evolution during battery charging and discharging. As shown in Figure S5, the interfacial resistance sharply increases to ∼1.2 × 10^4^ Ω after initial cycling. To identify the origins of this resistance growth, the distribution of relaxation times (DRT) analysis was employed to deconvolute the impedance spectra. As shown in Figures 1f and S6, the GEIS profiles of the LNMO ASSLBs can be decoupled into five characteristic peaks (marked as D_1_ to D_5_). The D_1_ and D_2_ peaks are assigned to Li^+^ transport across the Li‐In and LPSCl interlayer [27]. The D_3_ and D_4_ peaks originate from charge transfer resistance (*R*_ct_) at the cathode/LIC interface, while the D_5_ peak is ascribed to the solid‐phase diffusion (*R*_d_) within the cathode. Notably, both *R*_ct_ and *R*_d_ increase significantly during battery charging and discharging [28]. This pronounced impedance rise demonstrates that the formation of a substantial SCL severely impedes Li^+^ transportation at LNMO/LIC interface. Furthermore, the excessively high operating potential of LNMO cathodes induces parasitic reactions between the cathode and the LIC SE.

To elucidate the mechanism of this interfacial degradation, soft x‐ray absorption spectroscopy (sXAS) was employed to monitor the electronic structure evolution of the LNMO/LIC interface. As shown in Figure 1g, the O K‐edge spectra collected in total electron‐yield mode (TEY, detection depth ∼ 5–10 nm) reveal the surface‐sensitive chemical state of oxygen. The spectra are divided into two energy regions: the pre‐edge region below ∼534 eV reflecting strong hybridization between O 2p and TM 3d orbitals (split into t_2g_ and e_g_ states), and the region above ∼534 eV associated with O 2p and TM 4sp hybridization. In addition, a feature at 534 eV is attributed to surface carbonate species [29]. After cycling, the O K‐edge pre‐edge exhibits a pronounced redistribution characterized by a significant intensity decrease in the e_g_ peak. This indicates changes in TM–O hybridization [30]. The O K‐edge spectra obtained in total fluorescence yield (TFY) mode (probing depth ∼150–200 nm) exhibit negligible changes compared with TEY mode spectra (Figure S7), suggesting that oxygen instability is predominantly localized at the electrode surface or interfacial region.

Figure 1h shows the Mn L‐edge spectra, where the L_3_ and L_2_‐edges are indexed to electronic transitions from the Mn 2p_3/2_ and 2p_1/2_ core levels. After cycling, the Mn L‐edge spectra exhibit a distinct low‐energy feature at 639 eV assigned to Mn^2+^ species [31], indicating irreversible Mn reduction at the interfacial or near‐surface region. Similarly, the reduction of Ni is also observed after cycles, as validated by the obvious peak position shift of Ni L_3_‐edge (Figure S8). These reductions occur to compensate for the local charge following partial oxygen loss at the LNMO surface, reflecting severe structural collapse at the interface. To identify specific parasitic products at the LNMO/LIC interface, x‐ray photoelectron spectroscopy (XPS) was employed. As shown in Figure 1i, the pristine In 3d spectrum exhibits typical doublet peaks at 445.3 and 453.0 eV, corresponding to 3d_5/2_ and 3d_3/2_ split peaks for In^3+^ [32]. After cycling, a new pair of split peaks appears at 443.0 and 450.4 eV, indexed to metallic In^0^ [33]. The presence of In^0^ demonstrates the decomposition of LIC into non‐stoichiometric Li_3+_ *_x_* InCl_6_, LiCl, and metastable In–Cl species [14].

Consequently, the combination of SCL formation and oxygen‐related interfacial parasitic reactions under high voltage results in sluggish interfacial Li^+^ transport and performance decay. Achieving highly reversible LNMO ASSLBs therefore requires simultaneously suppressing the SCL effect and mitigating oxygen‐induced interfacial degradation.

### 2.2 Characterizations of BTO‐S‐LNMO Cathodes

**FIGURE 2:**
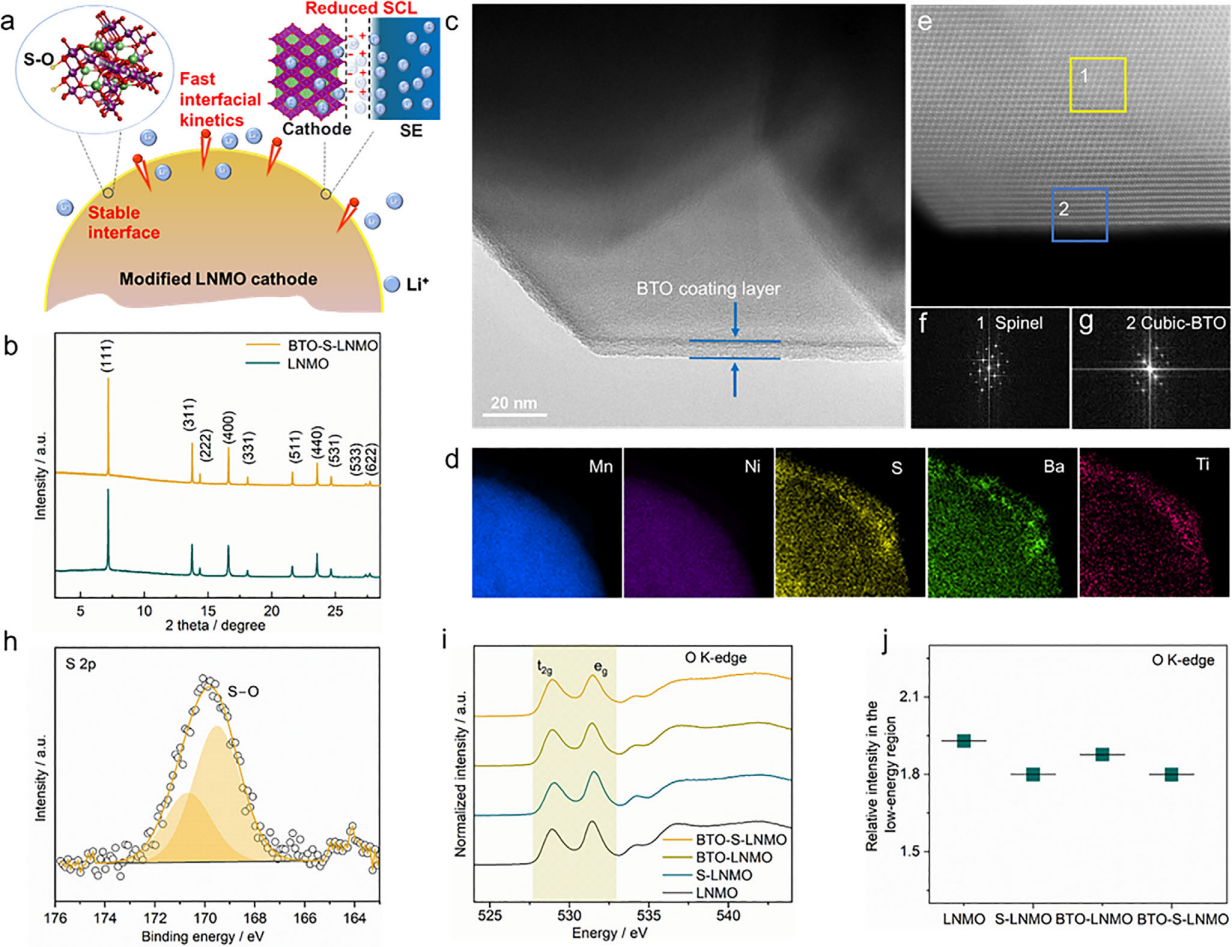
Schematic illustration and structural characterization of BTO‐S‐LNMO. (a) Schematic illustration of BTO‐S‐LNMO ASSLB. (b) Synchrotron XRD patterns of LNMO and BTO‐S‐LNMO samples. (c) The STEM images and (d) EDS mapping of BTO‐S‐LNMO samples. (e) HR‐TEM images and (f and g) the corresponding FFT patterns for the dotted regions. (h) S 2p XPS spectra of BTO‐S‐LNMO. (i) O K‐edge of all samples. (j) Variation of the integrated intensity in the low‐energy region (shaded region) in i for O K‐edge.

A coupled strategy involving interfacial potential regulation and anion‐chemistry stabilization was designed to address critical interfacial issues in LNMO ASSLBs. As illustrated in Figure 2a, piezoelectric BTO was introduced at the LNMO/SE interface to regulate the local potential distribution via a built‐in electric field to suppress SCL formation [34]. In addition, S─O bonds were induced via sulfidation to enhance oxygen stability and mitigate interfacial degradation. Synchrotron‐based x‐ray powder diffraction (XRD) patterns demonstrate that the main diffraction peaks of BTO‐S‐LNMO match well with those of the pristine LNMO (Figure 2b), confirming the well‐preserved spinel structure without impurity phases [35]. Scanning electron microscopy (SEM) image reveals that pristine LNMO possesses morphology of secondary spherical particles with a diameter of 2–4 µm composed of nano‐size primary particles (Figure S9a). After modification, the BTO‐S‐LNMO cathodes show similar morphology (Figure S9b), suggesting the modification has a negligible impact on the bulk structure.

To analyze the microstructure of cathodes, scanning transmission electron microscopies (STEM) of two cathodes were conducted. As shown in Figures 2c and S10 and S11, a uniform BTO coating layer with a thickness of ∼4 nm is established on the surface of BTO‐S‐LNMO cathode. The energy‐dispersive spectroscopy (EDS) mappings show that Ni and Mn are dominant in the bulk, while Ba, Ti, and S are enriched at the surface (Figure 2d), indicating the formation of BTO and interfacial sulfide layer. The high‐resolution TEM (HR‐TEM) of BTO‐S‐LNMO cathodes was performed to analyze the crystal structure (Figure 2e). The well‐resolved lattice fringes indicate the high crystallinity of the BTO‐S‐LNMO cathodes. The fast Fourier transform (FFT) pattern of region 1 (Figure 2e, marked by a yellow line) confirms the spinel phase of bulk LNMO (Figure 2f). In contrast, region 2 (Figure 2e, marked by a blue line) at the surface exhibits a cubic phase, corresponding to the BTO coating at the surface (Figure 2g). To further understand the interactions among S, BTO, and LNMO, XPS was employed to investigate the surface chemical states of BTO‐S‐LNMO. As shown in Figure S12, the strong split peaks in Ba 3d and Ti 2p spectra confirm the presence of BTO at the surface of BTO‐S‐LNMO. The S 2p spectra exhibit two characteristic peaks at 169.5 eV (S 2p_3/2_) and 170.6 eV (S 2p_1/2_) (Figures 2h and S13), indicating S─O bond formation [36]. Compared to the pristine LNMO, the O 1s spectrum of BTO‐S‐LNMO displays a pronounced additional peak at ∼532.7 eV (Figure S14), which further confirms oxygen species engaged in covalent S─O bonding [37, 38]. This result was further demonstrated by Fourier transform infrared spectroscopy (FTIR), which shows distinct vibrational bands associated with S─O stretching modes (1066∼1177 cm^−1^) (Figure S15) [39, 40]. These results demonstrate the successful introduction of the BTO coating with local S─O bridges on the LNMO surface. To decouple the individual contributions of BTO coating and S─O modulation, sulfurized LNMO (S‐LNMO) and BTO‐coated LNMO (BTO‐LNMO) were prepared by analogous procedures. sXAS and XAS were employed to analyze their electronic structures, oxidation states, and local coordination environments. As shown in Figure 2i,j, O K‐edge spectra were collected to probe the oxygen electronic structure. The integrated intensity of two characteristic peaks with t_2g_ and e_g_ in the low‐energy region is widely used as a qualitative indicator of the TM–O covalency [41]. Compared with LNMO, both S‐LNMO and BTO‐S‐LNMO exhibit a lower normalized pre‐edge intensity, suggesting weakened TM 3d–O 2p hybridization [42, 43]. Combined with the O 1s XPS analysis, this effect can be attributed to strong covalent S─O bonding, which is expected to downshift the O 2p energy level and consequently weaken TM–O hybridization. The reduced TM–O covalency is generally considered beneficial for stabilizing lattice oxygen under high voltage by mitigating O 2p band variations during electrochemical cycling [44, 45], thereby alleviating interfacial structural and chemical degradation. Mn K‐edge x‐ray absorption near‐edge structure spectroscopies (XANES) reveal that S‐LNMO and BTO‐S‐LNMO show distinct pre‐edge shift toward lower energy compared to pristine LNMO (Figure S16), suggesting a reduction of Mn oxidation state. While Mn valence of BTO‐LNMO remains unchanged compared to pristine LNMO, indicating that sulfidation drives the Mn charge compensation. Compared to pristine LNMO, the Ni K‐edge spectra reveal that BTO‐LNMO and BTO‐S‐LNMO exhibit a slight absorption edge shift toward lower energy (Figure S17), while S‐LNMO remains unchanged. This implies that the BTO coating layer influences the Ni electronic structure, potentially through interfacial polarization effect.

### 2.3 Electrochemical Performance of High‐Voltage BTO‐S‐LNMO ASSLBs

**FIGURE 3:**
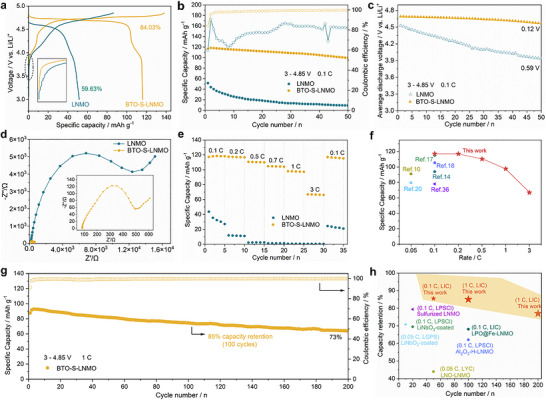
Electrochemical performance of high‐voltage BTO‐S‐LNMO ASSLBs. (a) Initial charge and discharge profiles of the two cathodes. (b) Cycling performances, (c) average discharge voltage of LNMO and BTO‐S‐LNMO ASSLBs at a rate of 0.1 C. (d) GEIS curve after cycling. (e) Rate capabilities. (f) Comparison of the rate capacity in LNMO ASSLBs between this work and reference data. (g) Long cycling performances at a high rate of 1 C. (h) Comparison of the cycle performance.

The electrochemical performances of ASSLBs with BTO‐S‐LNMO cathodes were evaluated. As shown in Figure 3a, the ASSLBs with BTO‐S‐LNMO cathodes present a notably extended charging plateau at ∼4.7 V compared to the pristine LNMO cathodes, suggesting activated Ni redox reactions. This improvement is further supported by the significant Ni redox peaks observed in the CV profiles (Figure S18). Furthermore, the initial CE of BTO‐S‐LNMO is 84.03%, significantly surpassing that of pristine LNMO (59.63%) and reflecting improved Ni redox reversibility. Accordingly, the ASSLB with the BTO‐S‐LNMO cathode delivers a significantly higher initial capacity of 116 mAh g^−1^ compared to the pristine LNMO cathode (52 mAh g^−1^), approaching the theoretical value of LNMO (147 mAh g^−1^).

The cycling performances of ASSLBs with pristine LNMO and BTO‐S‐LNMO cathodes were then compared. As shown in Figures 3b and S19, the ASSLBs with pristine LNMO cathodes suffer from rapid capacity loss, which only remains 9.2 mAh g^−1^ after 50 cycles. In contrast, the ASSLBs with BTO‐S‐LNMO cathodes showcase stable cycling performance at 0.1 C with a high‐capacity retention of 85.5% after 50 cycles, demonstrating superior interfacial stability. Control experiments with different BTO and S contents (Figure S20) further confirm the optimal performance. Besides, the ASSLBs with pristine LNMO suffer from a severe voltage drop of 0.59 V after 50 cycles, while this voltage drop is limited to 0.12 V when using BTO‐S‐LNMO cathodes (Figure 3c), further confirming the improved stability. The Nyquist plots further reveal that ASSLBs with BTO‐S‐LNMO cathodes retain an impedance of 483 Ω after 50 cycles, which is much lower than that of the pristine LNMO cathodes (∼1.2 × 10^4^ Ω) (Figure 3d). This confirms that the coupled strategy effectively accelerates Li^+^ migration across the interface, boosting the electrochemical performance.

The rate capability of ASSLBs with pristine LNMO and BTO‐S‐LNMO cathodes was then compared. As displayed in Figures 3e and S21, the BTO‐S‐LNMO cathode delivers specific capacities of 118.1, 117.3, 110.5, 104.6, 97.9, and 66.8 mAh g^−1^ at 0.1, 0.2, 0.5, 0.7, 1 and 3 C, respectively. In sharp contrast, the pristine LNMO cathode exhibits a low initial specific capacity at 0.1 C and fails to work under current densities exceeding 0.2 C. In comparison to the reported results, BTO‐S‐LNMO delivers high‐rate performance in ASSLB configuration (Figure 3f), verifying the effectiveness of SCL modulation and S─O bonding. Furthermore, the cycling stability of BTO‐S‐LNMO cathodes was evaluated at high charge/discharge rates. As shown in Figure 3g, ASSLBs with BTO‐S‐LNMO cathodes sustain stable cycling with 85% capacity retention after 100 cycles and 73% capacity retention after 200 cycles. A comprehensive comparison with the state‐of‐the‐art LNMO ASSLBs employing sulfide and halide SE systems highlights the significant achievement in rate capability and long‐term cycling stability of BTO‐S‐LNMO cathodes (Figure 3h and Table S1). These results demonstrate the effectiveness of the synergistic electro‐mechanical field regulation and anion‐chemistry stabilization strategy, underscoring the practical potential of LNMO for next‐generation high‐performance solid‐state energy storage devices.

### 2.4 Suppressed SCL and Boosted Interfacial Kinetics

**FIGURE 4:**
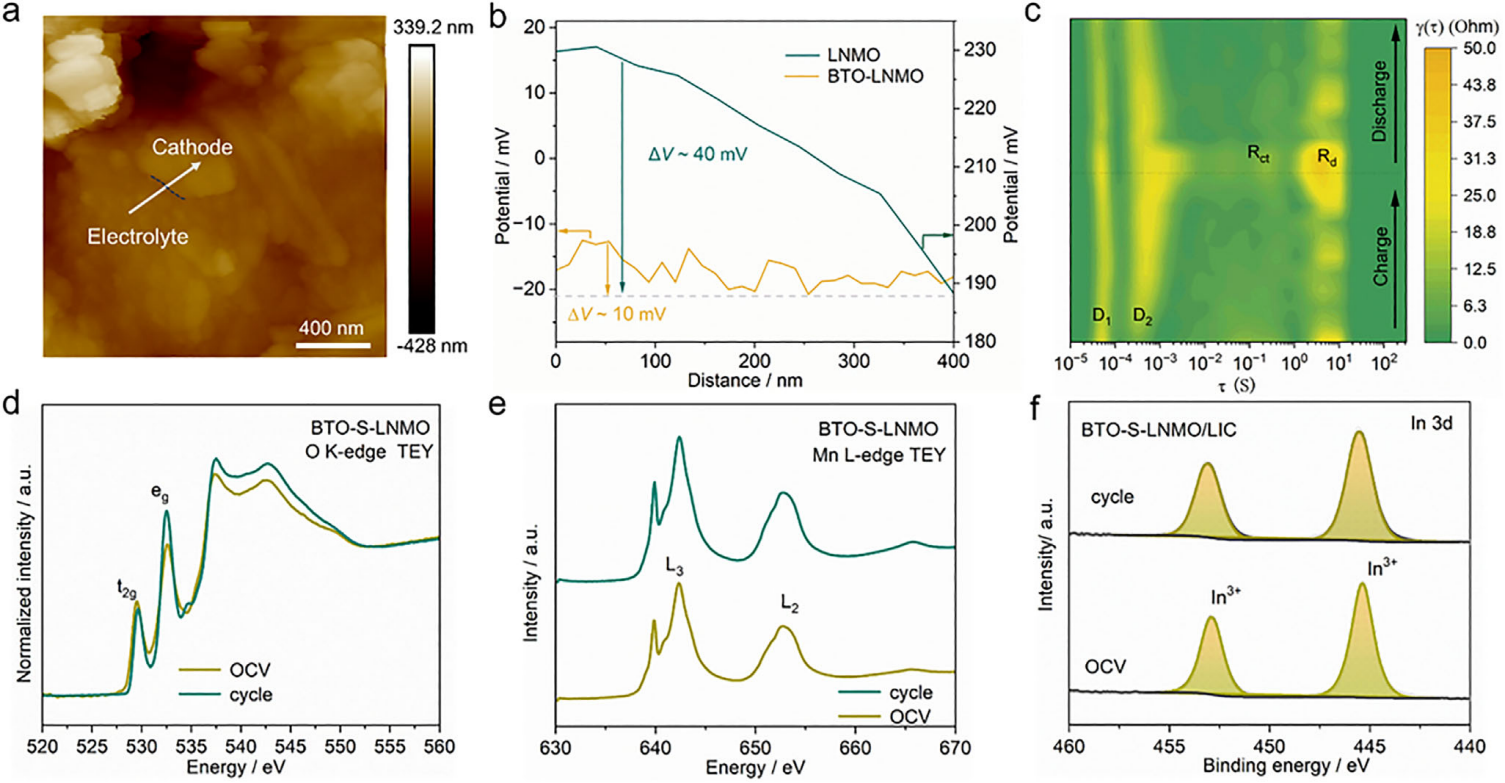
Potential distribution, kinetic properties, and chemical stability of BTO‐S‐LNMO/LIC interfaces. (a) AFM images of the BTO‐LNMO composite cathode. (b) Surface potential distribution from BTO‐LNMO to LIC by KPFM along the direction indicated by the straight line with arrows in (a). (c) DRT profile transformation derived from GEIS during the first cycle of BTO‐S‐LNMO ASSLBs. (d) O K‐edge, (e) Mn L‐edge for LNMO composite cathode and BTO‐S‐LNMO composite cathode before and after 50 cycles in the TEY mode. (f) In 3d XPS spectra of BTO‐S‐LNMO/LIC composite cathodes before and after 50 cycles.

To further validate the interfacial improvements, the ion transport behavior and electronic/chemical evolution at the interface were investigated. To elucidate the critical role of BTO in suppressing SCL formation, the AFM‐KPFM test was conducted on BTO‐LNMO to eliminate the influence of sulfate anions. As shown in Figure 4a,b, the interfacial potential difference between BTO‐LNMO and LIC is only 10 mV. This is substantially lower than the 40 mV observed at the pristine LNMO/LIC interface, demonstrating that the dielectric BTO layer effectively reduces the interfacial potential difference to mitigate the SCL. To ensure the reliability of the potential measurements, histograms of interfacial potential distribution obtained from bearing analysis were also provided. As shown in Figure S22, the BTO‐LNMO/LIC interface exhibits a significantly narrower distribution of 16 mV compared to 62 mV for the LNMO/SE interface. This result indicates that the incorporation of the piezoelectric BTO layer results in a flatter interfacial potential profile, thereby reducing the barrier for interfacial Li^+^ migration. Subsequently, in situ GEIS measurements combined with DRT analysis were conducted to evaluate Li^+^ transport kinetics at the interface in BTO‐S‐LNMO ASSLB. As shown in Figure S23, BTO‐S‐LNMO cathode exhibits minimal resistance variation. More importantly, the resistance increases during battery charging and decreases upon discharging, indicating improved interfacial kinetics and a highly reversible interfacial evolution process. DRT analyses (Figures 4c and S24) further reveal that both *R*_ct_ and *R*_d_ remain at low values throughout battery charging and discharging, verifying that the coupled interfacial regulation strategy effectively suppresses SCL formation to accelerate interfacial diffusion kinetics.

The sXAS of BTO‐S‐LNMO cathodes were conducted before and after cycling to track the interfacial electronic structure evolution. As shown in Figures 4d and S25, different from cycled pristine LNMO cathodes, the BTO‐S‐LNMO cathodes exhibit largely preserved O K‐edge pre‐edge line shapes in both the TEY and TFY detection modes, indicating that the TM–O hybridization framework is effectively maintained. Consistently, the Mn and Ni L‐edge spectra of BTO‐S‐LNMO are nearly identical before and after cycling, as shown in Figures 4e and S26. Given the high sensitivity of Mn and Ni L‐edge to the occupancy of Mn/Ni 3d states, the observed spectral stability indicates that BTO‐S‐LNMO possesses excellent redox reversibility. This enhanced interfacial chemical stability is further corroborated by XPS analysis, which reveals no detectable oxidative decomposition of the LIC SE after cycling (Figure 4f). These results demonstrate that the synergistic interfacial regulation effectively stabilizes both Li^+^ transport and interfacial chemistry, thereby enabling improved capacity retention and long‐term cycling stability in BTO‐S‐LNMO ASSLBs.

### 2.5 Stabilized Interphases and Structures of Cycled Cathode

**FIGURE 5:**
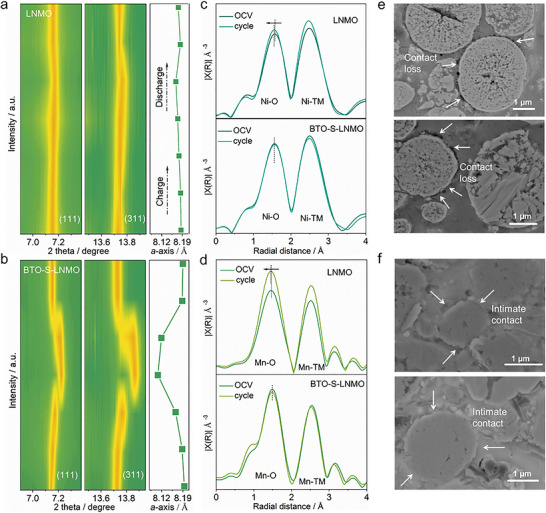
Alleviated interfacial degradation and improved structure reversibility of BTO‐S‐LNMO ASSLB. XRD phase evolution of the (111) and (311) reflections and corresponding lattice parameter evolution for (a) LNMO and (b) BTO‐S‐LNMO ASSLBs. FT‐EXAFS of (c) Ni K‐edge and (d) Mn K‐edge in LNMO and BTO‐S‐LNMO ASSLBs before and after 50 cycles. Focused ion beam‐scanning electron microscopy (FIB‐SEM) images of (e) LNMO/LIC and (f) BTO‐S‐LNMO/LIC composite cathodes.

*S*ynchrotron‐based XRD was performed to investigate the crystal structure stability of the cathodes during lithiation/delithiation. As shown in Figures 5a and S27, the pristine LNMO exhibits negligible (111) and (311) peak shifts during battery charging, suggesting limited Li^+^ intercalation/deintercalation. Furthermore, after the initial charge–discharge cycle, the (111) and (311) peaks exhibit a pronounced shift toward higher angles, indicating irreversible structural evolution, which is consistent with the low discharge capacity observed for LNMO in ASSLB (Tables S2 and S3). In contrast, BTO‐S‐LNMO exhibits well‐defined and reversible peak shifts throughout the charge/discharge process (Figure 5b). The average *a*‐axis lattice parameter smoothly decreased from 8.1944 to 8.1078 Å during charging and almost fully recovered to 8.1901 Å at the end of discharging, reflecting ideal lattice expansion‐contraction behavior (Tables S4 and S5). Such highly reversible lattice evolution indicates enhanced structural stability and effective utilization of the active phase, providing an important structural basis for the high rating capacity and cycling stability of BTO‐S‐LNMO in ASSLBs.

To investigate the local structural evolution of LNMO and BTO‐S‐LNMO, Mn and Ni K‐edges XAS measurements were collected for cycled LNMO and BTO‐S‐LNMO cathodes (Figures S28 and S29). The corresponding Fourier‐transformed extended x‐ray absorption fine structure (FT‐EXAFS) spectra derived from these XAS data are presented in Figure 5c,d. In these spectra, the first coordination‐shell peaks located at ∼1.5 Å are assigned to Mn─O and Ni─O bonding, reflecting the local octahedra environments of MnO_6_ and NiO_6_ [6]. It is noted that both Mn─O and Ni─O interatomic distances for LNMO cathodes shortened after cycling, indicating a distortion in the local MnO_6_ and NiO_6_ octahedral environments [46], likely resulting from oxygen instability and related degradation. In contrast, the Mn─O and Ni─O interatomic distances of BTO‐S‐LNMO remain unchanged after long‐term cycling, demonstrating a stable local coordination environment. These results confirm that BTO‐S‐LNMO possesses enhanced structural stability, ensuring more stable and reversible Li^+^ (de)intercalation over prolonged operation.

To further visualize interfacial degradation, FIB‐SEM was employed to examine the cross‐sectional morphology of the cycled composite cathodes. As shown in Figure 5e, the FIB‐SEM image of the cycled LNMO/LIC composite reveals pronounced interfacial gaps, indicating decomposition‐driven contact loss at the LNMO/LIC interface. In contrast, the BTO‐S‐LNMO composite cathode maintains intimate and continuous physical contact with the LIC after prolonged cycling, with no discernible interfacial gaps or contact loss (Figure 5f). Collectively, these observations demonstrate that BTO‐S‐LNMO not only stabilizes the crystal structure across multiple length scales but also effectively suppresses mechanically driven interfacial failure, thereby contributing to the superior electrochemical performance of BTO‐S‐LNMO ASSLBs.

## 3 Conclusion

In this study, we systematically investigated the interfacial kinetics and chemical evolution of high‐voltage LNMO ASSLB through AFM‐KPFM and GEIS‐DRT analyses. The sluggish interfacial Li^+^ transport at the LNMO/LIC interface is driven by SCL formation, resulting in high interfacial resistance and suppressing Ni redox activation, ultimately limiting the discharge capacity. Meanwhile, high‐voltage‐induced lattice oxygen instability triggers irreversible structural degradation at the LNMO/LIC interface, as evidenced by the attenuated pre‐edge features in the O K‐edge spectra. Mn and Ni L‐edges further reveal the formation and accumulation of reduced transition‐metal species, which hinder interfacial charge transfer and exacerbate performance degradation. To address these challenges, we develop a coupled electro‐mechanical field regulation and anion‐chemistry stabilization strategy. The spontaneous polarization of BTO generated an internal reverse electric field, effectively reducing the interfacial potential difference at the LNMO/LIC interface, weakening the SCL effect, and homogenizing the electric field distribution. Meanwhile, the sulfate‐derived S─O covalent bond weakens TM–O hybridization and stabilizes lattice oxygen, thereby suppressing oxygen‐driven structural and chemical degradation at the interface during cycling. These effects markedly enhanced Li^+^ transport kinetics and ensured sustained ion mobility over prolonged cycling, resulting in significantly improved electrochemical performance. This work demonstrates an effective strategy for enabling high‐voltage, long‐life ASSLBs and offers a versatile design principle applicable to next‐generation high‐energy solid‐state battery chemistry.

## Conflicts of Interest

The authors declare no conflicts of interest.

## Supporting information




**Supporting File 1**: Anie73047‐sup‐0001‐SuppMat.docx.

## Data Availability

The data that support the findings of this study are available from the corresponding author upon reasonable request.
