# Supplementary material for: Synergistic Regulation of Interfacial Potential and Anionic Covalency for High‐Voltage Cobalt‐Free All‐Solid‐State Batteries
Source: Angew Chem Int Ed Engl. 2026 Jun 4;65(32):e9550345. doi: 10.1002/anie.9550345 (PMC13427176; doi:10.1002/anie.9550345)
Supplement: Supplementary file 1 — Supporting File 1: Anie73047‐sup‐0001‐SuppMat.docx. [file ANIE-65-e9550345-s001.docx]

**Supporting Information**

**for**

**Synergistic Regulation of Interfacial Potential and Anionic Covalency for High-Voltage Cobalt-Free All-Solid-State Batteries**

Yue Wang^[a]^, Shuibin Tu^[a]^, Long Qian^[a]^, Shijie Xu^[a]^, Chao Ye*^[a]^, and Shi-Zhang Qiao*^[a]^

[a] School of Chemical Engineering, Adelaide University, Adelaide, SA 5005, Australia.

*Email: s.qiao@adelaide.edu.au; chao.ye@adelaide.edu.au

**Experimental Section**

**Fabrication of LiNi_0.5_Mn_1.5_O_4_ (LNMO)** **and sulfurized LNMO (S-LNMO)**

All chemicals were used as received without further purification. Pristine LNMO and S-LNMO spinel cathodes were synthesized *via* a facile solid-state method using Li_2_CO_3_ (99.99%, Sigma-Aldrich) and Ni_0.25_Mn_0.75_CO_3_ (lab-scale preparation) as raw materials, with powdered sulfur (S, 99.98%, Sigma-Aldrich) as the S source. The specific synthesis process is as follows. Stoichiometric amounts of Ni_0.25_Mn_0.75_CO_3_, Li_2_CO_3_ (5% excess), and powdered sulfur were mixed using an agate mortar for 1 h. The obtained mixture was subsequently calcined at 700 ℃ for 4 h and 915 ℃ for 12 h in air. After naturally cooling to room temperature, the LNMO and S-LNMO powders were obtained.

**Fabrication of** **BaTiO_3_**-**coated LNMO (BTO-LNMO) and BaTiO_3_**-**coated S-LNMO (BTO-S-LNMO)**

Citric acid was used as a chelating agent, butyl titanate as the titanium source, and barium acetate as the barium source. A certain amount of LNMO was dispersed uniformly in anhydrous ethanol and stirred continuously at room temperature. Butyl titanate and barium acetate, each at 1 mol% relative to LNMO, were dissolved separately in appropriate amounts of anhydrous ethanol and deionized water, respectively. Citric acid, at a molar ratio of 3:2 relative to the total metal cations, was dissolved in an appropriate amount of anhydrous ethanol. Subsequently, the citric acid ethanol solution, butyl titanate ethanol solution, and barium acetate aqueous solution were sequentially added dropwise into the uniformly stirred LNMO ethanol suspension. The mixture was stirred at 80 °C until completely dried. It was then dried in a vacuum oven at 120 °C for 12 h. After cooling, the resulting solid was ground into a uniform powder using a mortar and subjected to a second calcination in a muffle furnace at 700 °C for 8 h. After cooling to room temperature, LNMO and S-LNMO cathode materials uniformly coated with BTO were obtained.

**Characterization methods**

Synchrotron XRD (λ = 0.5903 Å), synchrotron-based XANES (data were processed using Athena software), and synchrotron-based FT-EXAFS were performed on beamlines at the Australian Synchrotron (Clayton), part of ANSTO. The STEM analysis was conducted using a FEI Titan G2 80-300 microscope at 300 kV equipped with a probe corrector. The XPS measurements were performed on a Thermo ESCALAB 250 system with a monochromatic Al Kα (1486.6 eV) X-ray source to investigate the relative content and chemical states of the elements. To ensure consistent testing conditions, the cathode materials and SE were first assembled into batteries and subsequently disassembled for XPS measurements. The XPS data were fitted with the asymmetric Gaussian-Lorentzian sum function in Advantage software. The contact potential difference of LNMO and LIC in composite cathodes was measured by an atomic force microscope (AFM, Bruker Dimension Icon) with Kelvin probe force microscopy (KPFM) in an argon-filled glovebox. The bearing analysis was performed using NanoScope Analysis software in the selected area, which contained the outer boundary between the LNMO particle and the LIC SE. The potential values are discussed relative to the highest potential point within the selected area. The average potential within the selected region was determined by Gaussian fitting in Origin.

**Electrochemical characterizations**

Laboratory-scale all-solid-state batteries (10 mm inner diameter) were fabricated using composite cathodes composed of the synthesized cathode materials, LPSCl (≤5 μm, Zhongke guneng, Technology Co., Ltd), LIC (Shenzhen Kejing) and vapor-grown carbon fiber (VGCF, Showa Denko K.K., Japan) as the conductive additive. The cathode composite was prepared by mixing the active material, LIC, and VGCF in a weight ratio of 65:32:3 using an agate mortar for 1 h. All assembly processes were conducted in an argon-filled glovebox. A total of 80 mg of LPSCl SE was used as the separator layer. First, the SE layer was prepared by pressing 80 mg of LPSCl and 80 mg of LIC under 240 MPa for 2 min. Subsequently, a total of 5-7 mg of cathode composite powder was uniformly spread on one side of the SE layer and pressed at 360 MPa for 30 s to form the cathode layer. On the opposite side of the SE layer, a 50 μm-thick indium foil (~27 mg, 10 mm in diameter) was placed, followed by a 30 μm-thick lithium foil (~0.83 mg, 8 mm in diameter) onto the indium to serve as the Li source. The full cell assembly was encapsulated in a stainless-steel plate case and subjected to a constant pressure of ~20 MPa to ensure interfacial contact. Galvanostatic charge-discharge tests and cycling performance of assembled ASSLBs were evaluated at 27 ℃ using a standard battery testing instrument (Neware battery test system, CT-4008T-5V50mA-164, Shenzhen, China). All tests were performed at 3.0 - 4.85 V vs. Li/Li^+^.

**Supplemental Figures**

**
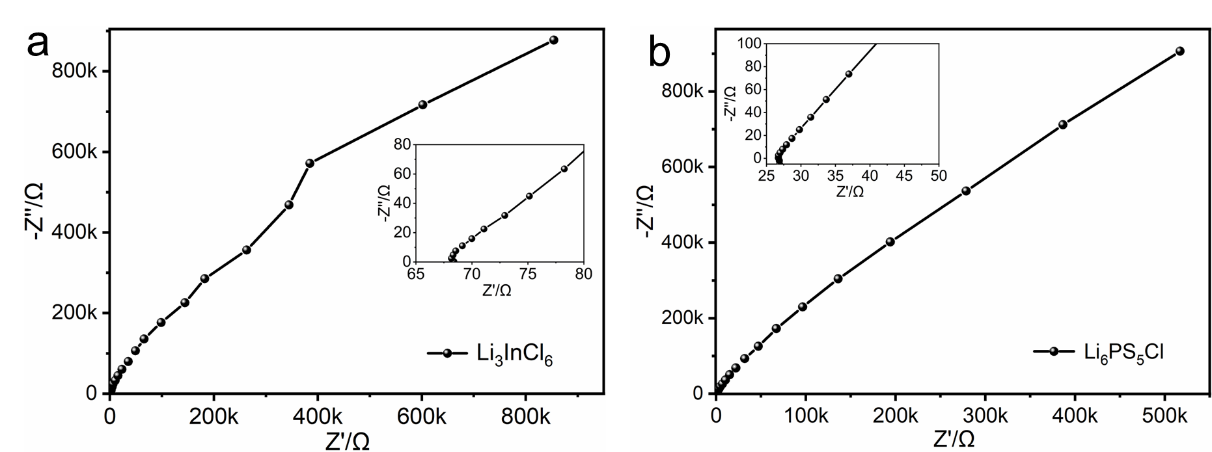
**

**Figure S1.** Nyquist plot of (a) LIC solid electrolyte and (b) LPSCl solid electrolyte.

Samples were prepared by densifying 100 mg of SE in a 10 mm diameter PEEK casing by 360 MPa for 3 min. With stainless steel rods as contacts, the measurement was performed in a frequency range from 1 MHz to 10 mHz with an excitation amplitude of 10 mV. As shown in **Figure S1**, the resistance of LIC and LPSCl are 68 Ω and 26 Ω, respectively. The SE pellet thicknesses are 629 μm and 930 μm, respectively. Therefore, according to the ionic conductivity equation $\sigma=\frac{L}{RA}$, where A is the sample's base area; L is the thickness; R is the Ohmic resistance. The calculated ionic conductivities of LIC and LPSCl are 1.18 and 4.55 mS cm^-1^, respectively.


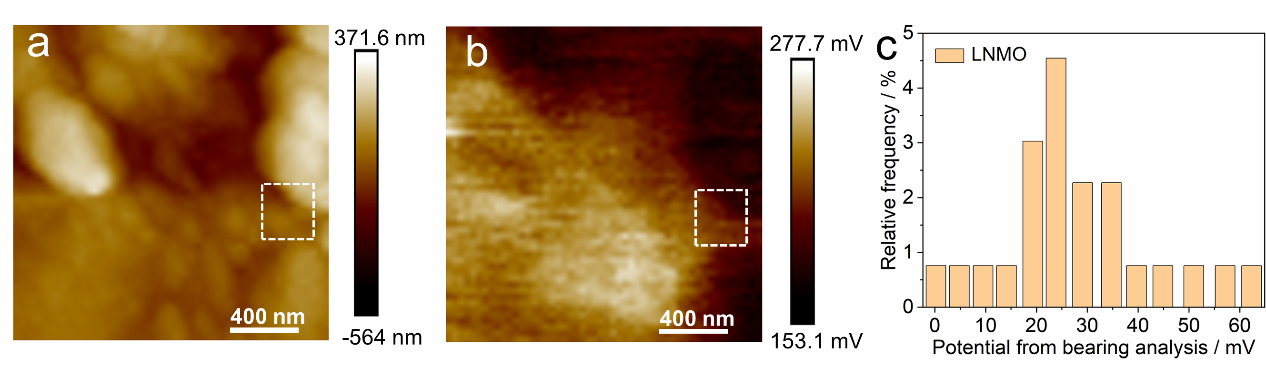


**Figure S2.** (a) The surface morphology and (b) surface potential of the LNMO composite cathode obtained by AFM and KPFM. The white line box exhibits the interfacial region between the LNMO and LIC. (c) A bearing analysis of the white line box in (b).

**
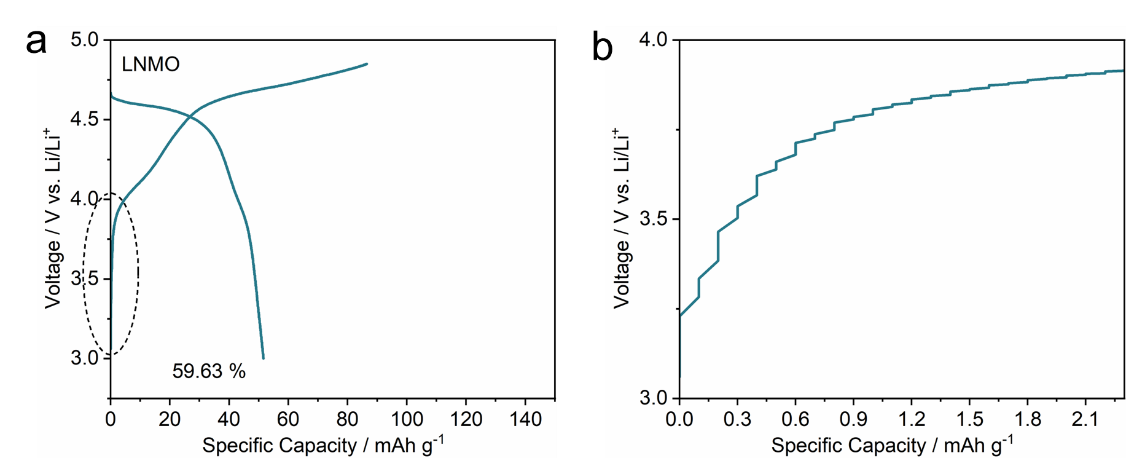
**

**Figure S3.** (a) Initial charge and discharge profiles of LNMO ASSLB, (b) enlarged view of the region highlighted by the dashed box in (a).


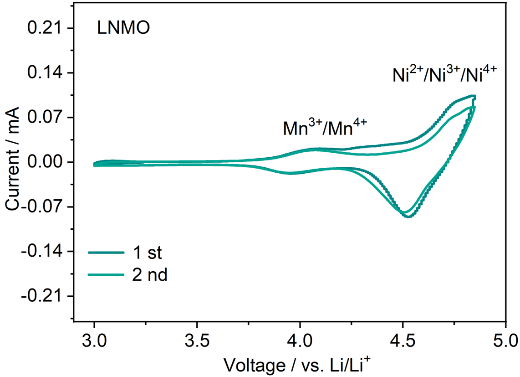


**Figure S4.** CV curves of ASSLB with pristine LNMO cathode.


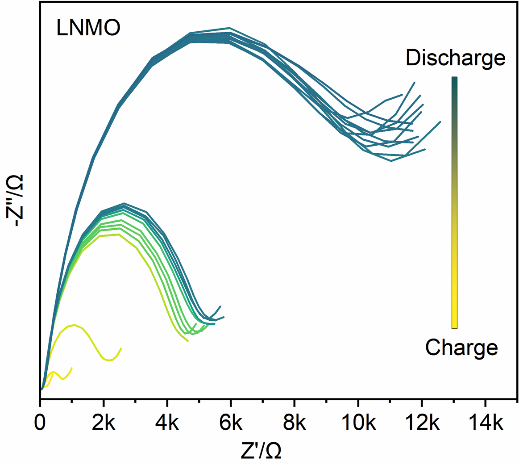


**Figure S5.** *In situ* impedance spectra during the first cycle of LNMO ASSLB.


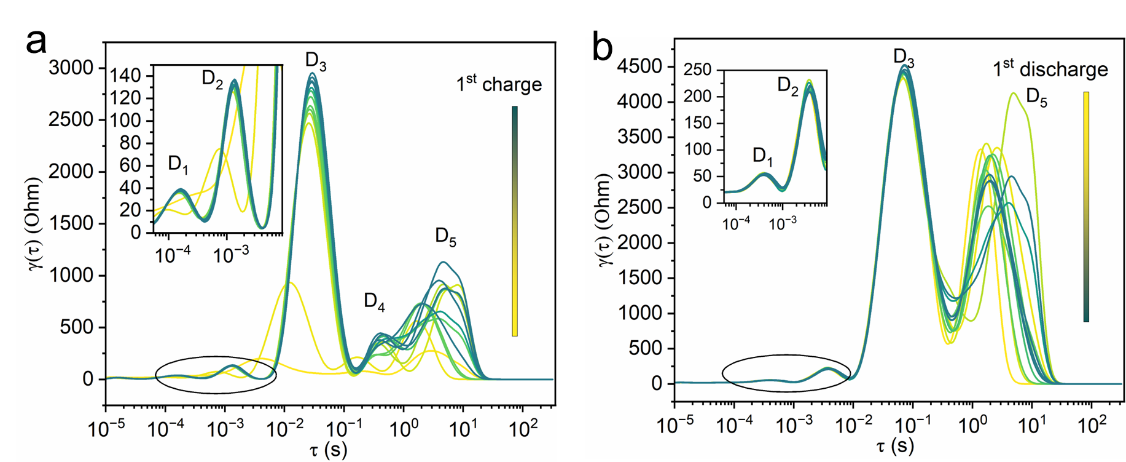


**Figure S6.** Evolution of DRT profile transformation derives from GEIS of LNMO ASSLB during (a) charging and (b) discharging process, highlighting interfacial resistance dynamics.


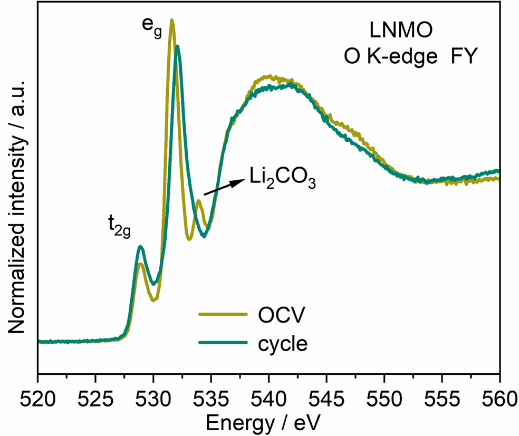


**Figure S7.** O K-edge for the LNMO composite cathode before and after 50 cycles in TFY mode.


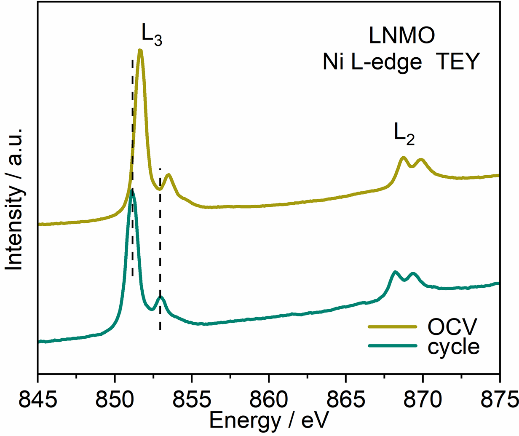


**Figure S8.** Ni L-edge for LNMO composite cathode before and after 50 cycles in the TEY mode.


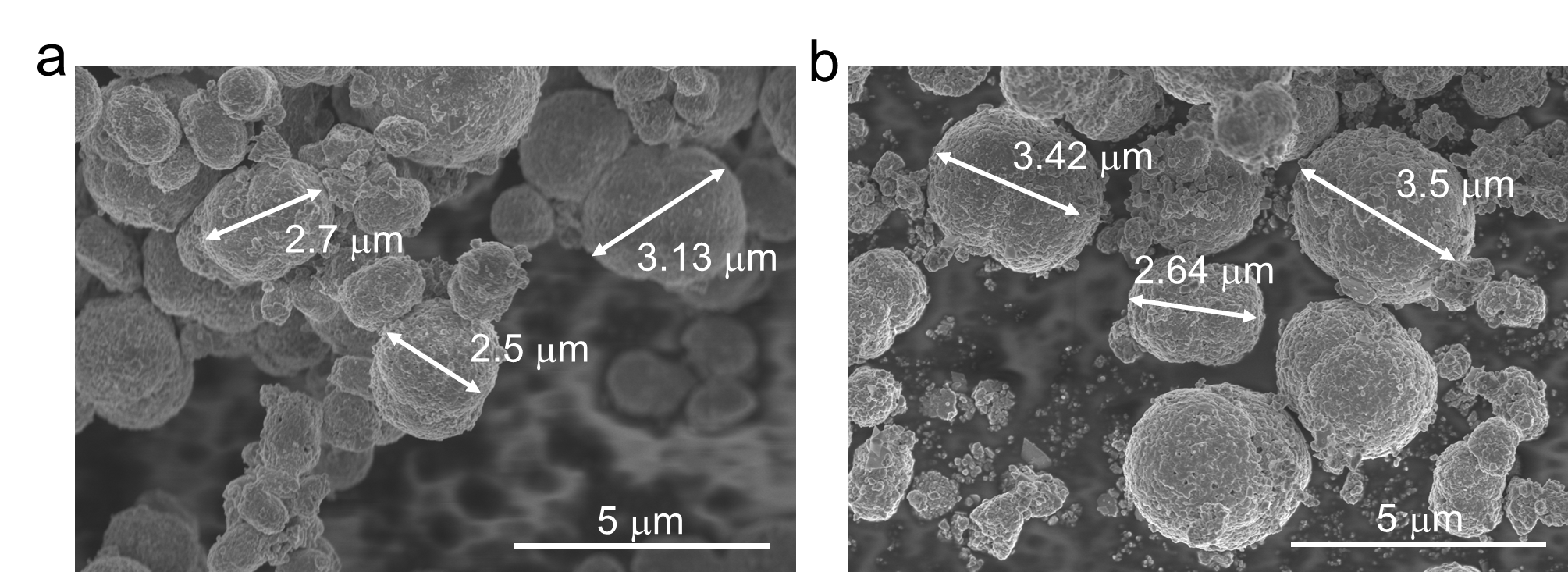


**Figure S9.** SEM images of (a) LNMO, (b) BTO-S-LNMO.


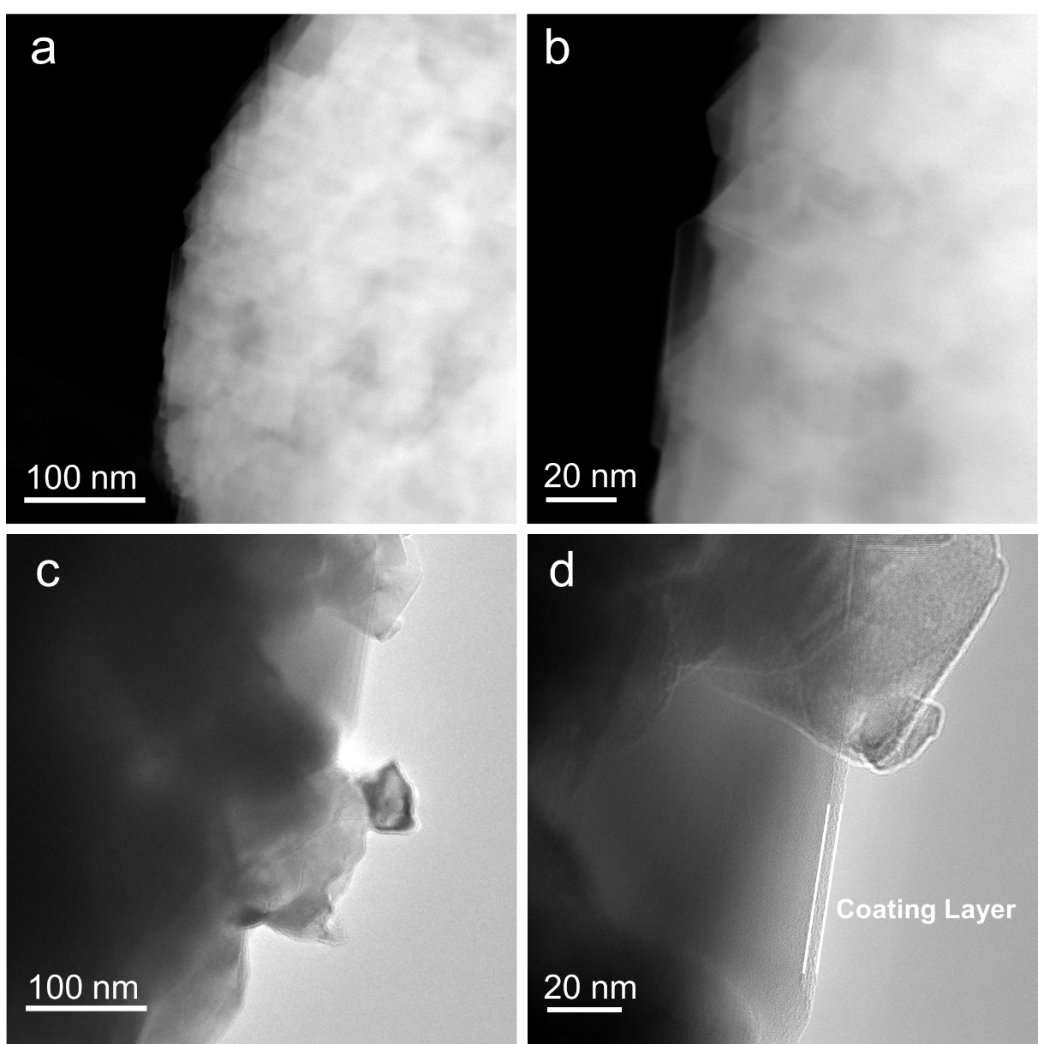


**Figure S10.** The STEM images of (a, b) LNMO and (c, d) BTO-S-LNMO.


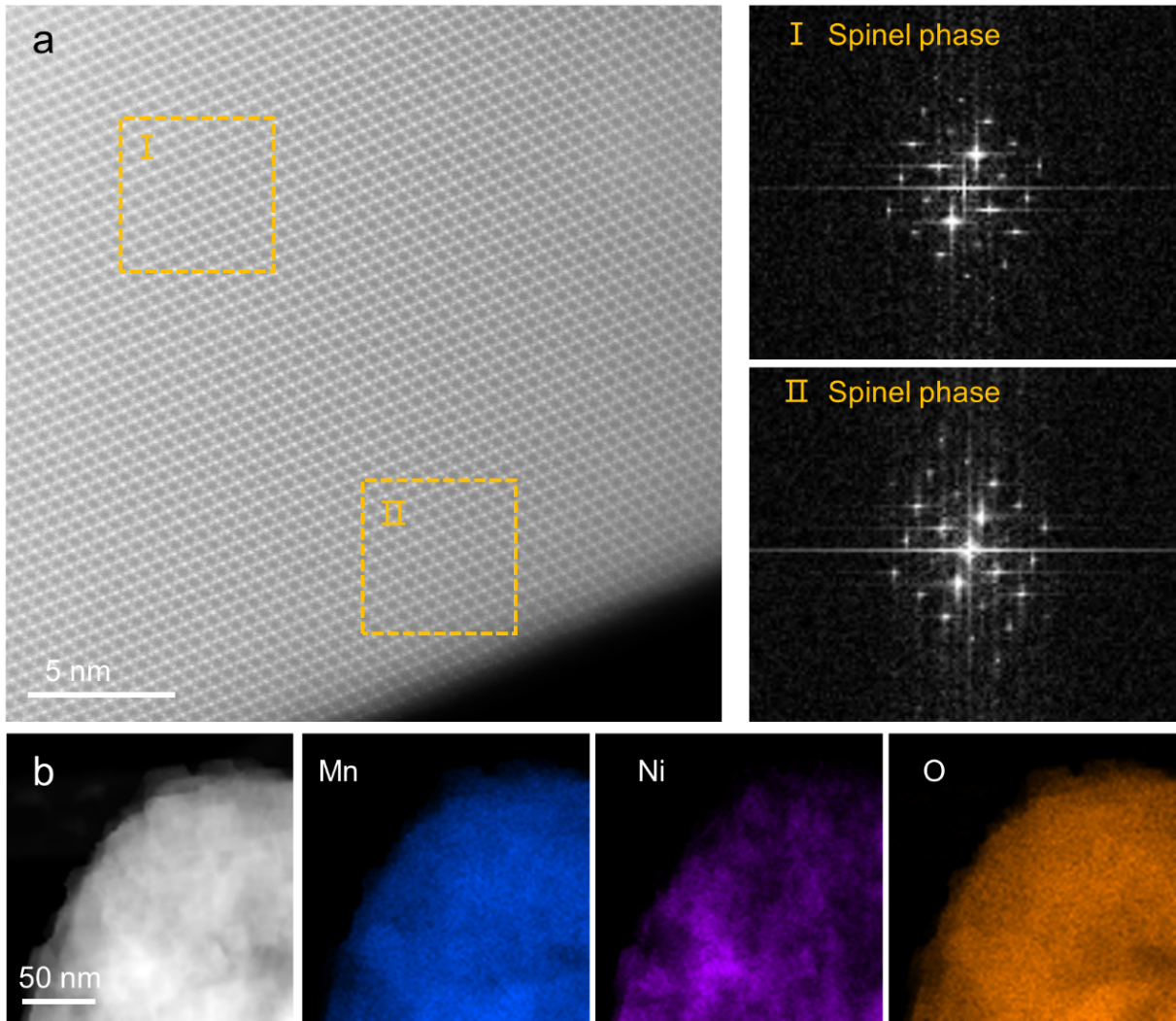


**Figure S11.** Morphology and structural characterization of LNMO. (a) The HR-TEM images of LNMO and the corresponding FFT pattern for dotted regions. (b) EDS mapping of LNMO.

HR-TEM was performed to analyze the crystal information of LNMO cathodes. As shown in **Figure S11**, well-resolved lattice fringes are observed in both the bulk and surface regions, indicating the high crystallinity of the LNMO cathodes. The FFT pattern for the selected region shows a spinel-phase structure, suggesting the well-preserved LNMO structure. In addition, EDS mappings reveal a homogeneous distribution of Ni, Mn and O elements throughout the LNMO particles.


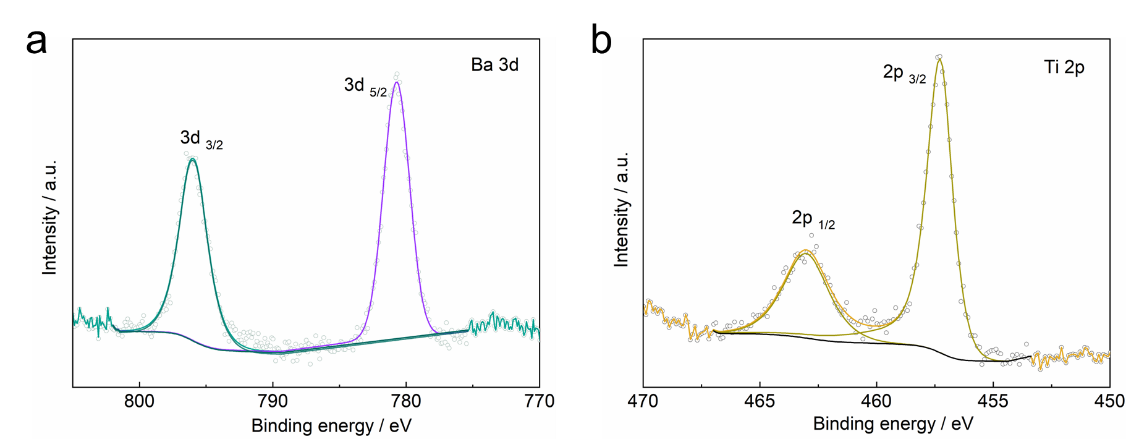


**Figure S12.** (a) Ba 3d, (b) Ti 2p XPS spectra of BTO-S-LNMO.

The Ba 3d XPS spectrum exhibits two well-defined peaks at 780.7 and 796 eV, corresponding to Ba 3d_5/2_ and Ba 3d_3/2_, respectively. The Ti 2p XPS spectrum displays distinct peaks at 457.3 and 463 eV, corresponding to Ti 2p_3/2_ and Ti 2p_1/2_, respectively.


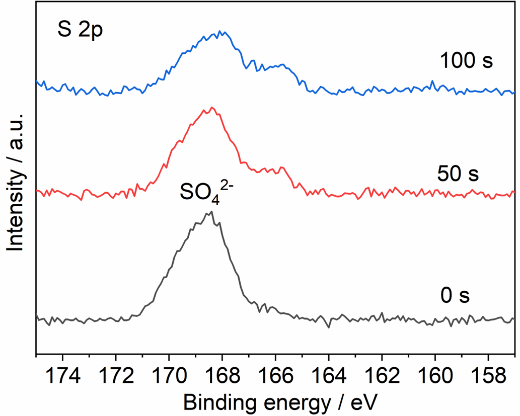


**Figure S13.** S 2p XPS depth profiles of BTO-S-LNMO cathodes.


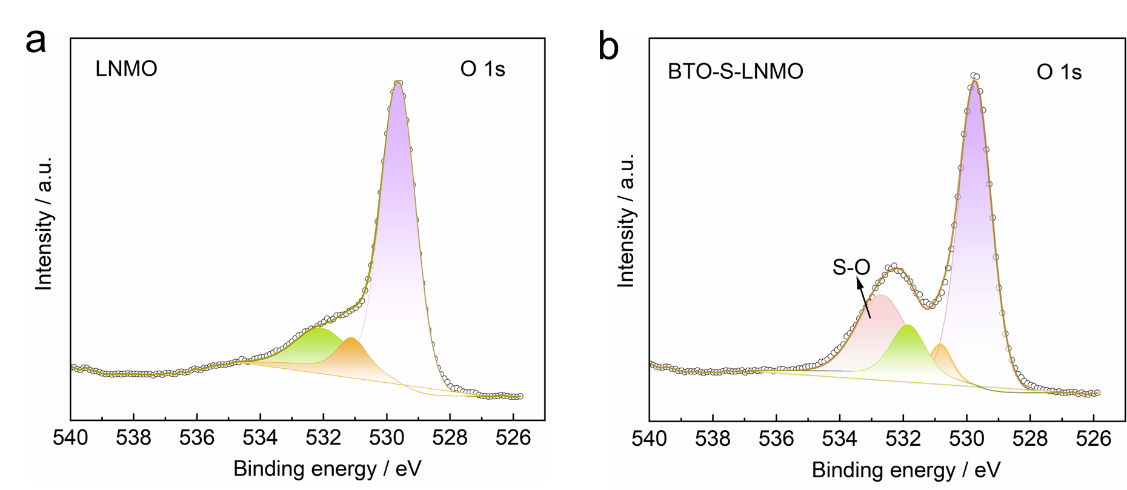


**Figure S14.** O 1s XPS spectra of (a) LNMO and (b) BTO-S-LNMO.


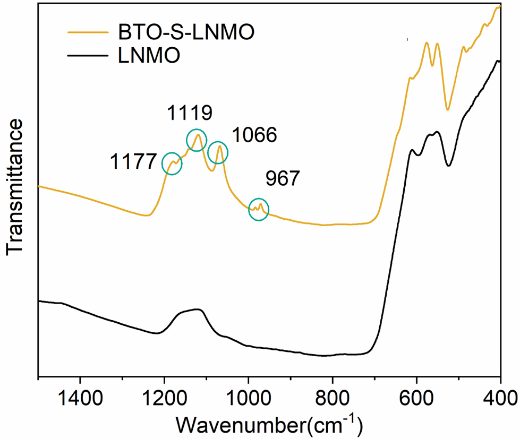


**Figure S15.** FTIR spectra of LNMO and BTO-S-LNMO.


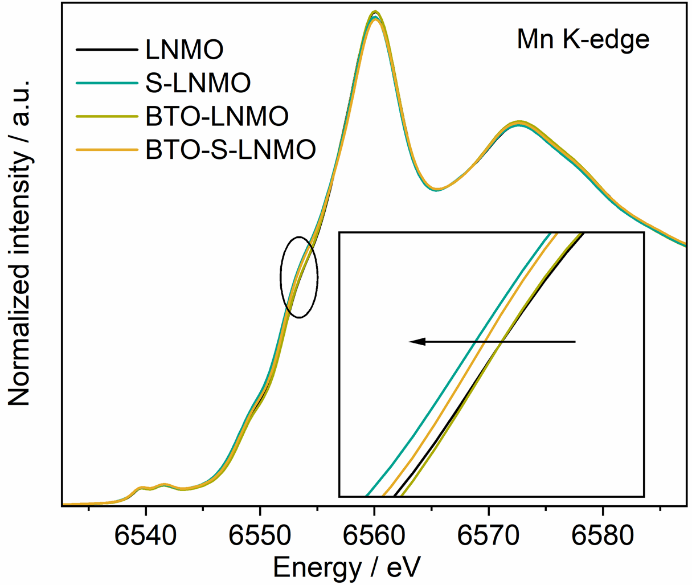


**Figure S16.** Mn K-edge of the LNMO, S-LNMO, BTO-LNMO and BTO-S-LNMO samples.


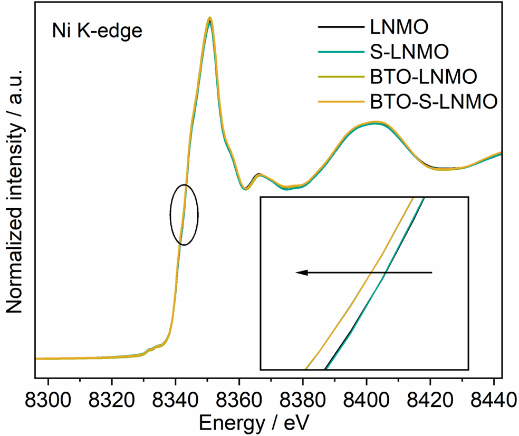


**Figure S17.** Ni K-edge of the LNMO, S-LNMO, BTO-LNMO and BTO-S-LNMO samples.


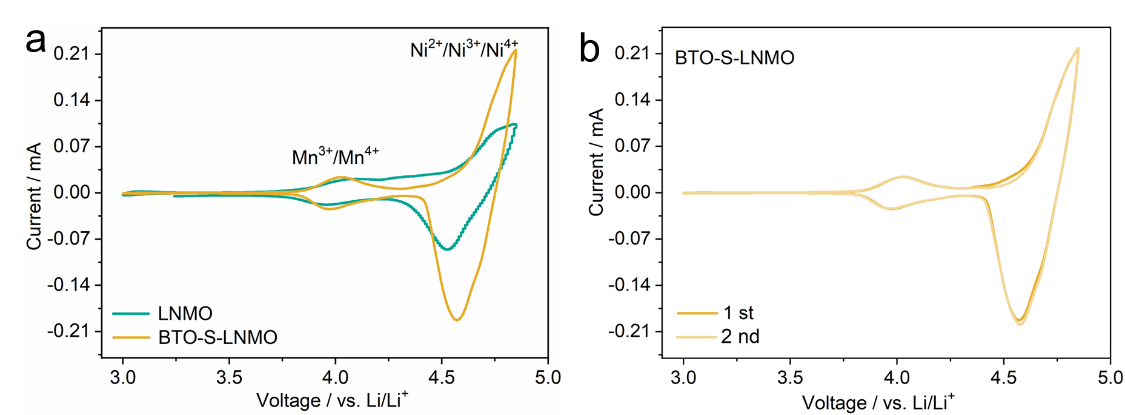


**Figure S18.** (a) Comparison of the first-cycle CV curves between the LNMO and BTO-S-LNMO ASSLBs. (b) CV curves for the first two cycles of BTO-S-LNMO.


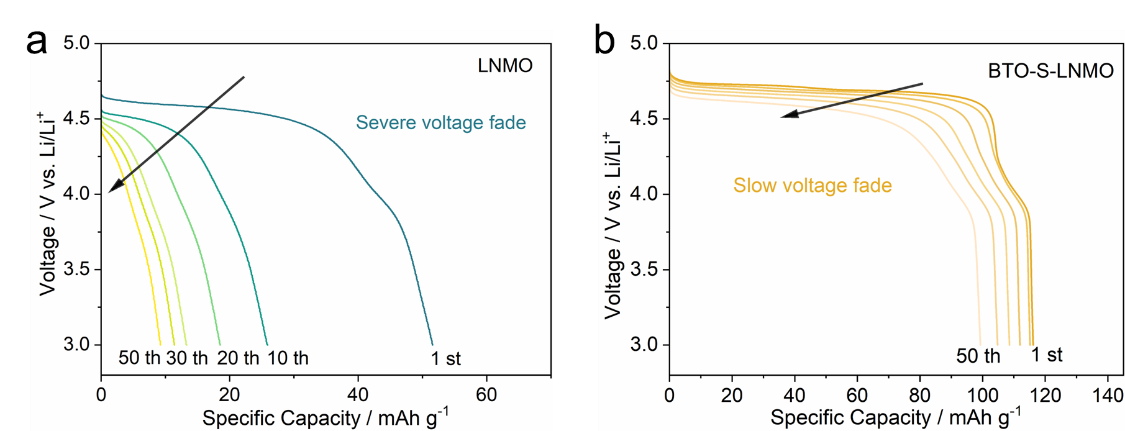


**Figure S19**. Discharge curves of ASSLBs with (a) LNMO and (b) BTO-S-LNMO cathodes at 0.1C.


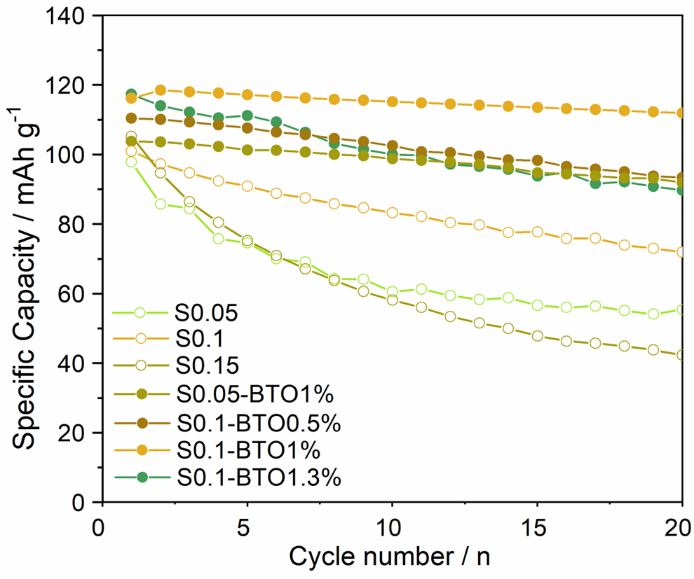


**Figure S20.** The cycling performances of different amounts of BTO and S in halide-based ASSLBs at 0.1 C.

The S content was varied in the range of 0.05-0.15 (mass ratio). The sample with S = 0.1 shows the best overall performance, while lower S content leads to limited improvement, and higher S content results in faster capacity decay. Based on this, the BTO content was further tuned from 0.5% to 1.3% (mass ratio). Among these, 1% of BTO delivers the highest capacity and the most stable cycling behavior, whereas both lower and higher BTO contents lead to inferior performance.


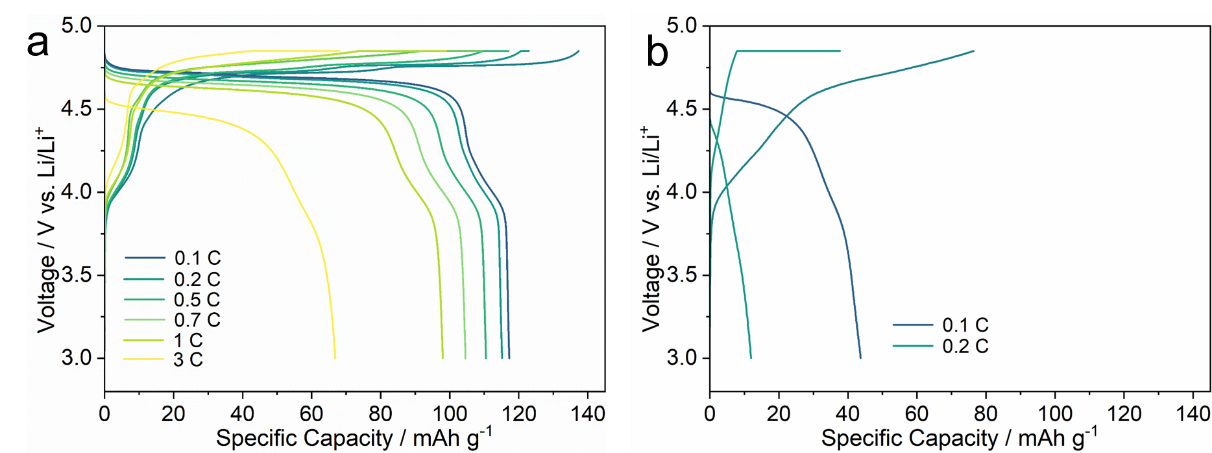


**Figure S21.** The charge-discharge curves of ASSLBs with (a) BTO-S-LNMO cathode and (b) LNMO cathode at different C-rates.


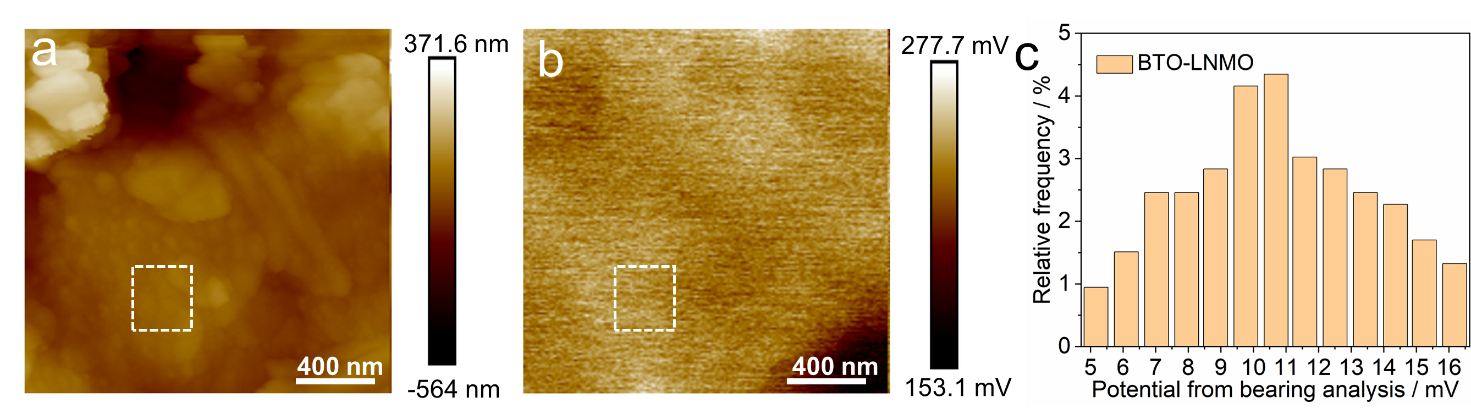


**Figure S22.** (a) The surface morphology and (b) surface potential of the BTO-LNMO composite cathode obtained by AFM and KPFM. The white line box exhibits the interfacial region between the LNMO and LIC. (c) A bearing analysis of the white line box in (b).


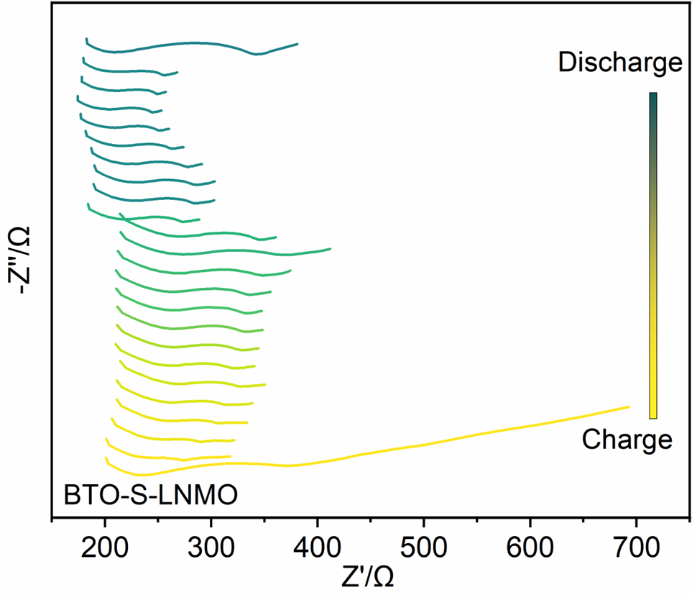


**Figure S23.** *In situ* impedance spectra during the first cycle of BTO-S-LNMO ASSLB.


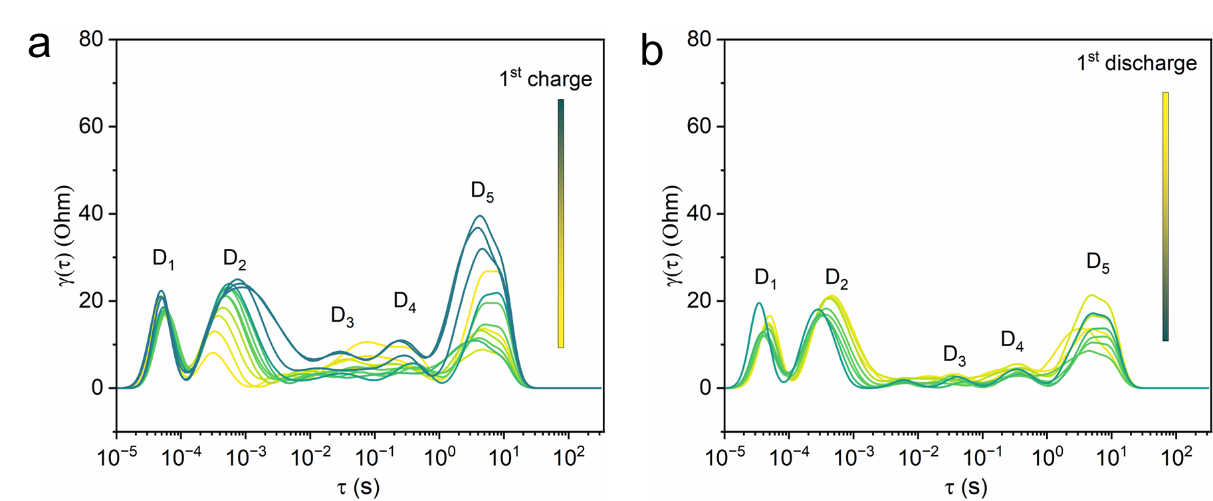


**Figure S24.** Evolution of DRT profile transformation derives from GEIS of BTO-S-LNMO ASSLB during (a) charging process and (d) discharging process.


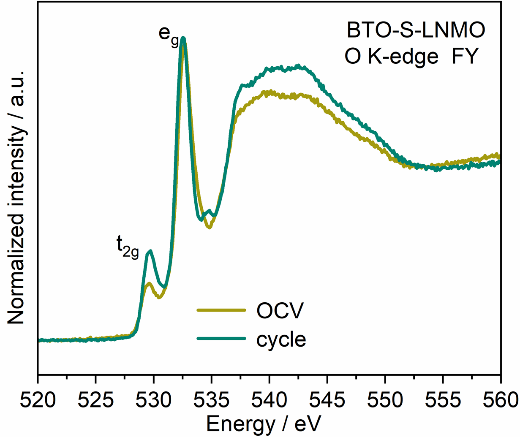


**Figure S25.** O K-edge for the BTO-S-LNMO composite cathode before and after 50 cycles in TFY mode.


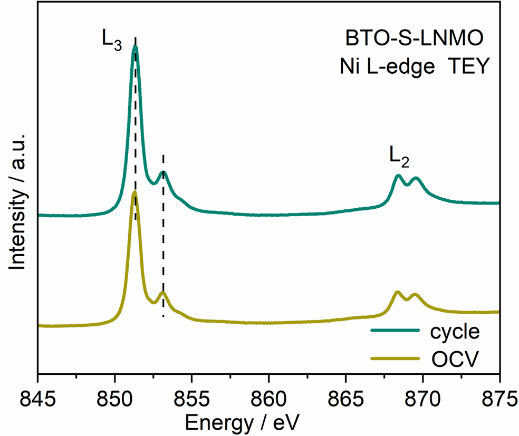


**Figure S26.** Ni L-edge for BTO-S-LNMO composite cathode before and after cycling in TEY mode.


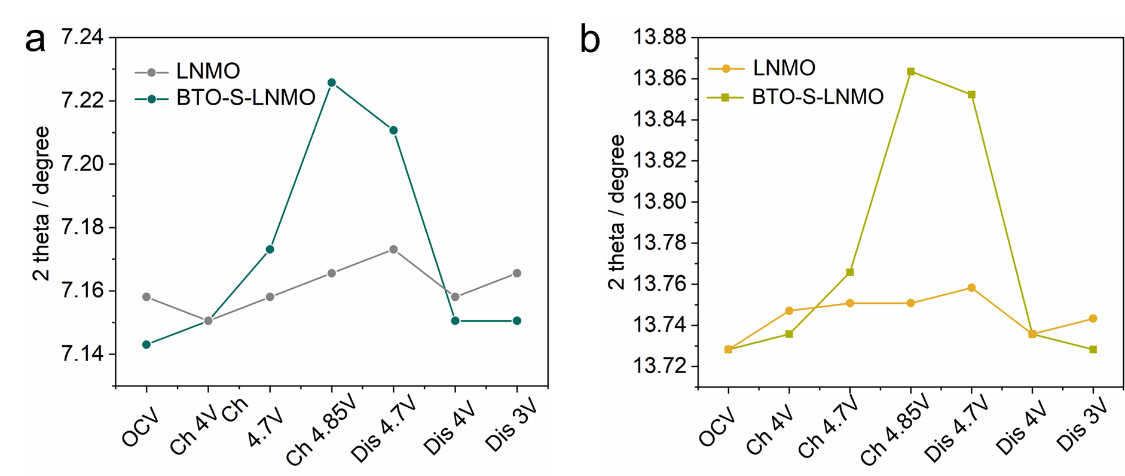


**Figure S27.** Diffraction peaks position variation of (a) (111) diffraction peaks and (b) (311) diffraction peaks.


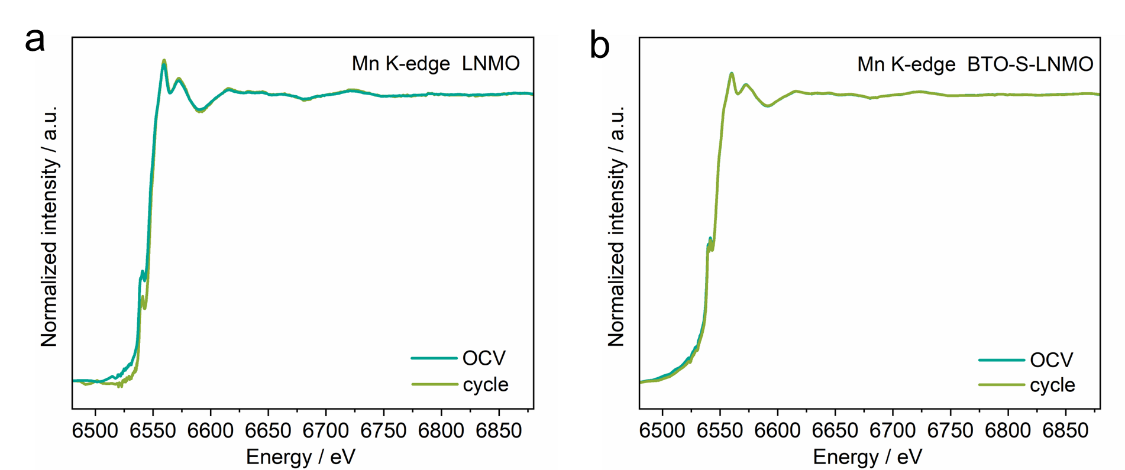


**Figure S28.** Mn K-edge for (a) LNMO composite cathode and (b) BTO-S-LNMO composite cathode before and after cycling.


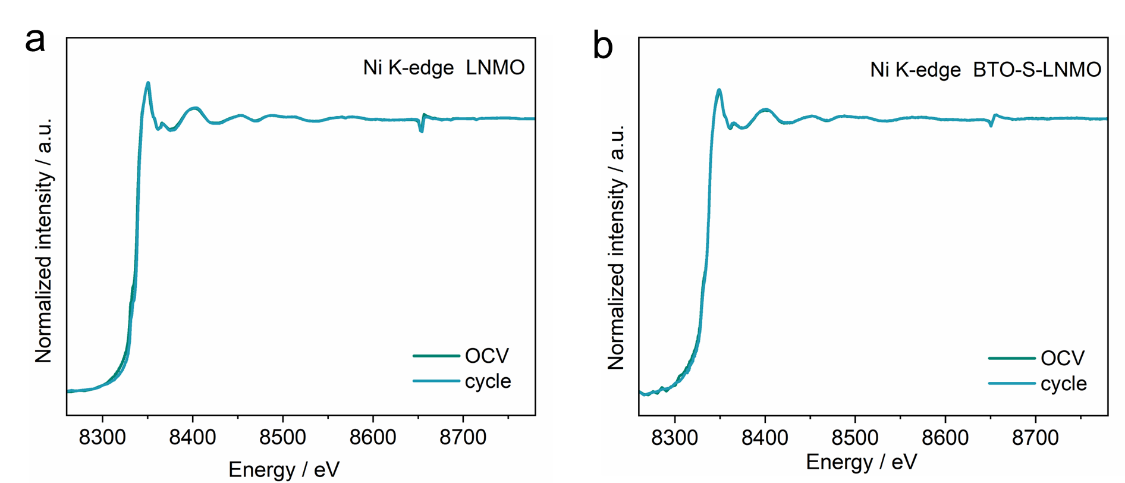


**Figure S29.** Ni K-edge for (a) LNMO composite cathode and (b) BTO-S-LNMO composite cathode before and after cycling.

**Table S1.** Capacity and cycling performance of state-of-the-art LNMO cathodes in ASSLBs**.**

| **Cathodes** | **Temperature**  **(℃)** | **SE** | **Current density (C)** | **Active material ratio** | **Initial capacity (mAh g^−1^)** | **Cycling performance** | **Ref.** |
| --- | --- | --- | --- | --- | --- | --- | --- |
| BTO-S-LNMO |  | LIC | 0.1 | 65:32:3 | 116.2 | 85% after 50 cycles | This work |
|  | 27 |  | 1 |  | 87.6 | 85% after 100 cycles |  |
|  |  |  |  |  |  | 73% after 200 cycles |  |
| Li_3_PO_4_-LNMO | 25 | LPSCl |  | 30:70:6 | 62 | none | 19 |
| LiNbO_3_-LNMO | / | LPSCl | 0.05 | 40:60:4 | 80 | none | 16 |
| LiNbO_3_-LNMO | 25 | LGPS | 0.05 | 38.5:57.5:4 | 80 | 71% after 10 cycles | 20 |
| LiNbO_3_-LNMO | / | LPSCl | 0.1 | 70:30 | 115 | 69.5% after 20 cycles | 17 |
| Sulfurized LNMO | 27 | LPSCl | 0.1 | 65:30:5 | 78 | 79.4% after 20 cycles | 36 |
| Al_2_O_3_-H-LNMO | 30 | LPSCl | 0.1 | 40:55:5/70:25:5 | 105.5 | 62.1% after 100 cycles | 18 |
| LNO-LNMO | 30 | LYC | 0.05 | 66:31:3 | 91 | 44% after 50 cycles | 10 |
| LPO@Fe-LNMO | / | LIC | 0.1 | 65:32:2:1. | 94 | 68.1% after 100 cycles | 14 |

Note: Li_10_GeP_2_S_12_ (LGPS), Li_3_YCl_6_ (LYC).

**Table S2.** The (111) and (311) diffraction peaks position variation in the initial charging/discharging process of LNMO-based ASSLB.

| **State of charge** | **Peak positions for (111)** | **Peak positions for (311)** |
| --- | --- | --- |
| OCV | 7.1581° | 13.7283° |
| Charge to 4 V | 7.1506° | 13.7471° |
| Charge to 4.7 V | 7.1581° | 13.7508° |
| Charge to 4.85 V | 7.1656° | 13.7508° |
| Discharge to 4.7 V | 7.1731° | 13.7583° |
| Discharge to 4 V | 7.1581° | 13.7358° |
| Discharge to 3 V | 7.1656° | 13.7433° |

**Table S3.** Evolution of *a*-axis in the initial charging/discharging process of LNMO-based ASSLB.

| **State of charge** | ***a*-axis from (111) diffraction peak** | ***a*-axis from (311) diffraction peak** | **Average *a*-axis** |
| --- | --- | --- | --- |
| OCV | 8.1852 Å | 8.1863 Å | 8.1858 Å |
| Charge to 4 V | 8.1938 Å | 8.1752 Å | 8.1845 Å |
| Charge to 4.7 V | 8.1852 Å | 8.173 Å | 8.1791 Å |
| Charge to 4.85 V | 8.1767 Å | 8.1730 Å | 8.1748 Å |
| Discharge to 4.7 V | 8.1681 Å | 8.1685 Å | 8.1683 Å |
| Discharge to 4 V | 8.1852 Å | 8.1819 Å | 8.1836 Å |
| Discharge to 3 V | 8.1767 Å | 8.177 Å | 8.1770 Å |

**Table S4.** The (111) and (311) diffraction peaks position variation in the initial charging/discharging process of BTO-S-LNMO-based ASSLB.

| **State of charge** | **Peak positions for (111)** | **Peak positions for (311)** |
| --- | --- | --- |
| OCV | 7.1430° | 13.7283° |
| Charge to 4 V | 7.1505° | 13.7358° |
| Charge to 4.7 V | 7.1731° | 13.7658° |
| Charge to 4.85 V | 7.2257° | 13.8635° |
| Discharge to 4.7 V | 7.2106° | 13.8523° |
| Discharge to 4 V | 7.1505° | 13.7358° |
| Discharge to 3 V | 7.1505° | 13.7283° |

**Table S5.** Evolution of *a*-axis in the initial charging/discharging process of BTO-S-LNMO-based ASSLB.

| **State of charge** | ***a*-axis from (111) diffraction peak** | ***a*-axis from (311) diffraction peak** | **Average *a*-axis** |
| --- | --- | --- | --- |
| OCV | 8.2025Å | 8.1863 Å | 8.1944 Å |
| Charge to 4 V | 8.1938 Å | 8.1819 Å | 8.1878 Å |
| Charge to 4.7 V | 8.1681 Å | 8.1641 Å | 8.1661Å |
| Charge to 4.85 V | 8.1087 Å | 8.1069 Å | 8.1078 Å |
| Discharge to 4.7 V | 8.1256 Å | 8.1134 Å | 8.1195 Å |
| Discharge to 4 V | 8.1938 Å | 8.1819 Å | 8.1878 Å |
| Discharge to 3 V | 8.1938 Å | 8.1863 Å | 8.1901 Å |
